# Tibial spino–meniscal clearance: A reproducible MRI‐derived measurement associated with hypermobile lateral meniscus

**DOI:** 10.1002/jeo2.70660

**Published:** 2026-02-16

**Authors:** Camilo Helito, Francisco Endara Urresta, Carlos Peñaherrera‐Carrillo, Alejandro Barros Castro

**Affiliations:** ^1^ Universidad de Sao Paulo Sao Paulo Brazil; ^2^ Clinica Arthros Quito Ecuador; ^3^ Instituto Nacional de Rehabilitacion Mexico City Mexico; ^4^ Universidad Internacional del Ecuador Quito Ecuador

**Keywords:** biomarker, hypermobility, knee, meniscus, MRI

## Abstract

**Purpose:**

To evaluate whether tibial spino–meniscal clearance (TSMC), a magnetic resonance imaging (MRI)‐derived imaging measurement, is associated with arthroscopically confirmed hypermobile lateral meniscus (HML), and to assess the reproducibility of this measurement.

**Methods:**

A combined retrospective–prospective diagnostic accuracy study was conducted including 164 patients who underwent knee MRI followed by arthroscopy within six months. TSMC was measured on sagittal MRI as the shortest distance between the apex of the lateral tibial spine and the inner margin of the posterior horn of the lateral meniscus. Three blinded observers independently performed measurements to assess intra‐ and interobserver reliability using intraclass correlation coefficients (ICCs). Diagnostic performance was evaluated using receiver operating characteristic (ROC) analysis, and multivariable logistic regression was used to examine the association between TSMC and HML.

**Results:**

Mean TSMC values were lower in patients with arthroscopically confirmed HML compared with controls. The measurement demonstrated excellent intra‐ and interobserver reliability. ROC analysis showed good discriminatory performance for identifying HML, and TSMC remained independently associated with hypermobility after adjustment for demographic and anatomical variables.

**Conclusion:**

TSMC is a reproducible MRI‐derived imaging measurement associated with HML. While these findings suggest that TSMC may assist in preoperative suspicion of lateral meniscal instability, further external validation is required before routine clinical application.

**Level of Evidence:**

Level III.

## Graphical abstract

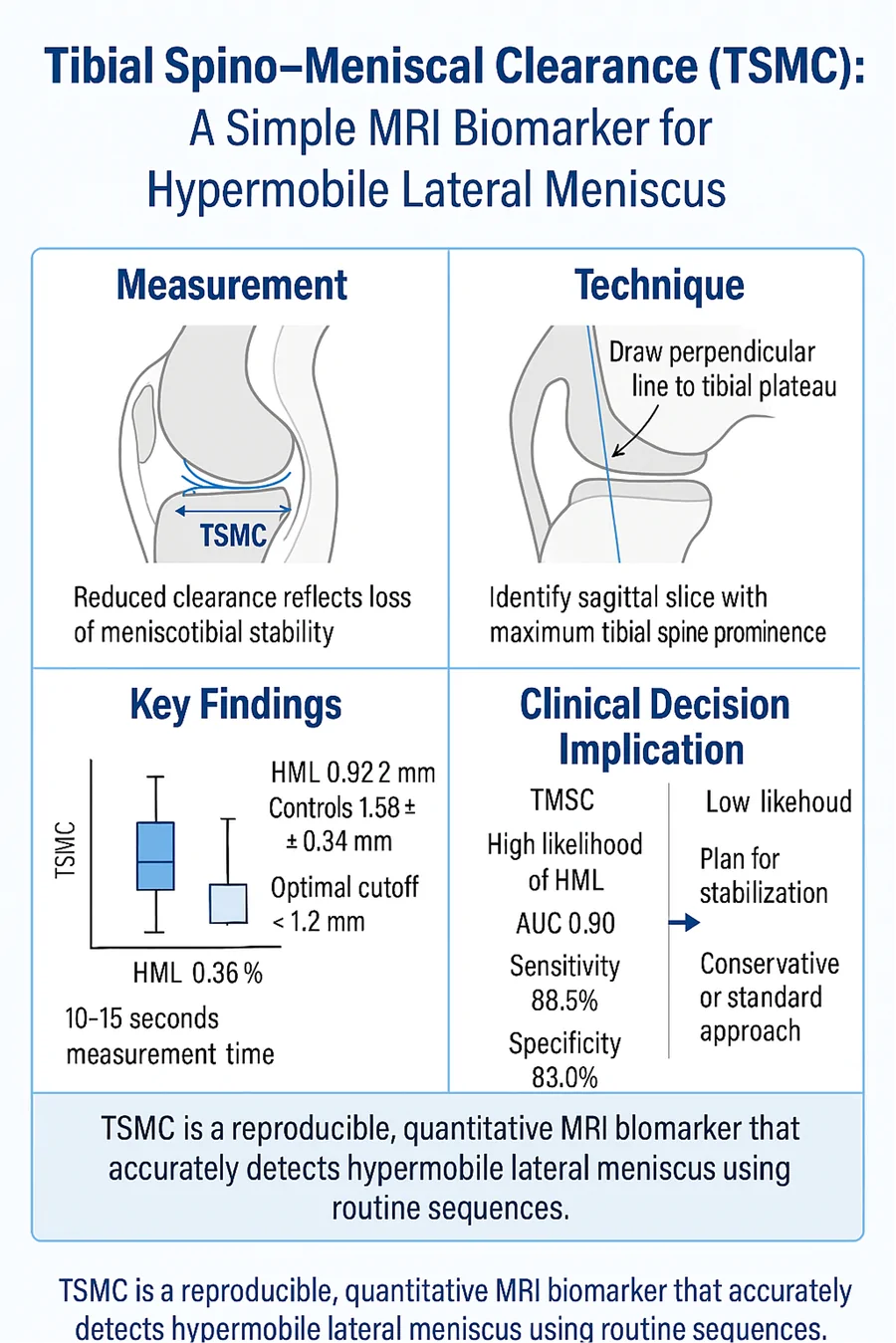

**Abbreviations**
ACL: anterior cruciate ligament
AUC: area under the curve
CI: confidence interval
HML: hypermobile lateral meniscus
ICC: intraclass correlation coefficient
MRI: magnetic resonance imaging
OR: odds ratio
ROC: receiver operating characteristic
TSMC: tibial spino–meniscal clearance

## INTRODUCTION

Hypermobile lateral meniscus (HML) remains an under‐recognized contributor to persistent lateral knee pain and rotational instability. Although the entity has been described for more than two decades, its diagnosis still depends almost exclusively on dynamic arthroscopic evaluation. Preoperative magnetic resonance imaging (MRI), despite being the standard modality for evaluating meniscal pathology, frequently fails to reveal signs of hypermobility. In routine clinical care, patients with atraumatic or recurrent lateral joint line symptoms often present with normal‐appearing MRI scans, leading to delayed diagnosis, inappropriate conservative treatment or partial meniscectomy of tissue that is structurally intact but biomechanically unstable. This diagnostic gap persists even as the understanding of meniscotibial ligament insufficiency and posterior horn instability has expanded in the arthroscopic literature [2, 4, 8].

The limitations of conventional MRI for detecting HML are primarily related to the fact that hypermobility is a functional phenomenon assessed under dynamic probing, whereas MRI is typically acquired in a neutral, unloaded position. Proposed indirect MRI findings—such as subtle posterior horn displacement or perimeniscocapsular fluid—are inconsistently present and show limited diagnostic performance. As a result, patients may present with persistent lateral joint‐line symptoms despite a ‘normal‐appearing’ MRI, leading to delayed diagnosis, inappropriate conservative management or unnecessary partial meniscectomy of structurally intact tissue. Improving preoperative identification of lateral meniscal instability is therefore clinically important to support timely diagnosis and appropriate meniscal‐preserving surgical planning [1, 10, 11].

The tibial spino–meniscal clearance (TSMC) arises from a straightforward anatomical rationale. The lateral tibial spine serves as a stable osseous landmark adjacent to the posterior horn of the lateral meniscus. Under normal conditions, the posterior horn maintains a consistent spatial relationship with the spine through intact meniscotibial attachments. When these stabilizing structures are lax or disrupted, as in HML, the posterior horn demonstrates excessive anteroposterior excursion, which should be reflected as a measurable reduction in the clearance between the meniscus and the tibial spine on routine sagittal MRI. The concept is mechanically intuitive: diminished clearance suggests abnormal mobility and laxity, whereas preserved clearance reflects stable fixation. Unlike extrinsic signs, TSMC focuses directly on the spatial relationship most relevant to meniscal stability [1, 4, 8, 10].

Because the measurement relies on a single linear distance, assessed on standard MRI slices without specialized sequences or dynamic imaging, TSMC has the potential to offer a simple, reproducible and clinically accessible biomarker. If validated against arthroscopic findings, it may allow surgeons to anticipate hypermobility, counsel patients more accurately and plan for meniscal stabilization techniques—such as posterior horn repair or meniscotibial ligament augmentation—rather than performing unnecessary partial meniscectomy.

The objective of this study is twofold: first, to determine whether TSMC measured on routine MRI can predict arthroscopically confirmed HML; and second, to evaluate the intra‐ and interobserver reproducibility of this measurement. We hypothesized that TSMC would be significantly lower in patients with HML and would demonstrate high reliability across observers, supporting its use as a practical preoperative diagnostic tool.

## METHODS

### Study design

This study was conducted as a diagnostic accuracy investigation that incorporated both retrospective and prospective components. The retrospective cohort consisted of patients who had undergone knee MRI at our institution and subsequently underwent arthroscopic evaluation within a six‐month interval, enabling a direct comparison between preoperative MRI findings and intraoperative assessment of lateral meniscal stability. The prospective cohort included patients scheduled for arthroscopy on the basis of clinical symptoms consistent with lateral meniscal pathology; these individuals underwent MRI using the same standardized imaging protocol prior to surgery. Combining both cohorts allowed for an expanded sample size while ensuring homogeneity in eligibility criteria, imaging techniques and arthroscopic assessment across the entire study period. Arthroscopy served as the reference standard for confirming HML. All procedures were performed by fellowship‐trained knee surgeons using a consistent probing protocol to evaluate posterior horn stability. Operative findings in the retrospective cohort were extracted from electronic records, while in the prospective cohort, they were documented using a structured case report form. Institutional ethics approval was obtained, and written informed consent was required for participation in the prospective arm.

### Patient selection

Eligibility required that patients had undergone an MRI at our institution according to a standardized protocol and subsequently underwent diagnostic or therapeutic knee arthroscopy within 6 months of imaging. Only individuals between 15 and 55 years of age were included to minimize the influence of age‐related degenerative changes in the meniscus. Complete imaging and operative information were mandatory for inclusion, and prospective participants provided written consent.

In the retrospective cohort, indications for arthroscopy were based on persistent mechanical knee symptoms refractory to conservative treatment, including lateral or medial joint‐line pain, mechanical catching or locking and clinical suspicion of meniscal pathology. This cohort, therefore, included patients undergoing arthroscopy for a heterogeneous set of indications, such as suspected medial meniscal tear, suspected lateral meniscal tear or unexplained lateral compartment symptoms. HML was not an a priori surgical indication in the retrospective cohort but was diagnosed intraoperatively during systematic arthroscopic assessment of posterior horn stability.

Patients were scheduled for arthroscopy due to persistent lateral compartment symptoms suggestive of meniscal pathology, including mechanical pain or instability unresponsive to nonoperative management. While HML was clinically suspected in these cases, definitive diagnosis and indication for stabilization were confirmed intraoperatively. No patient underwent surgery solely on the basis of MRI findings.

**Figure 1:**
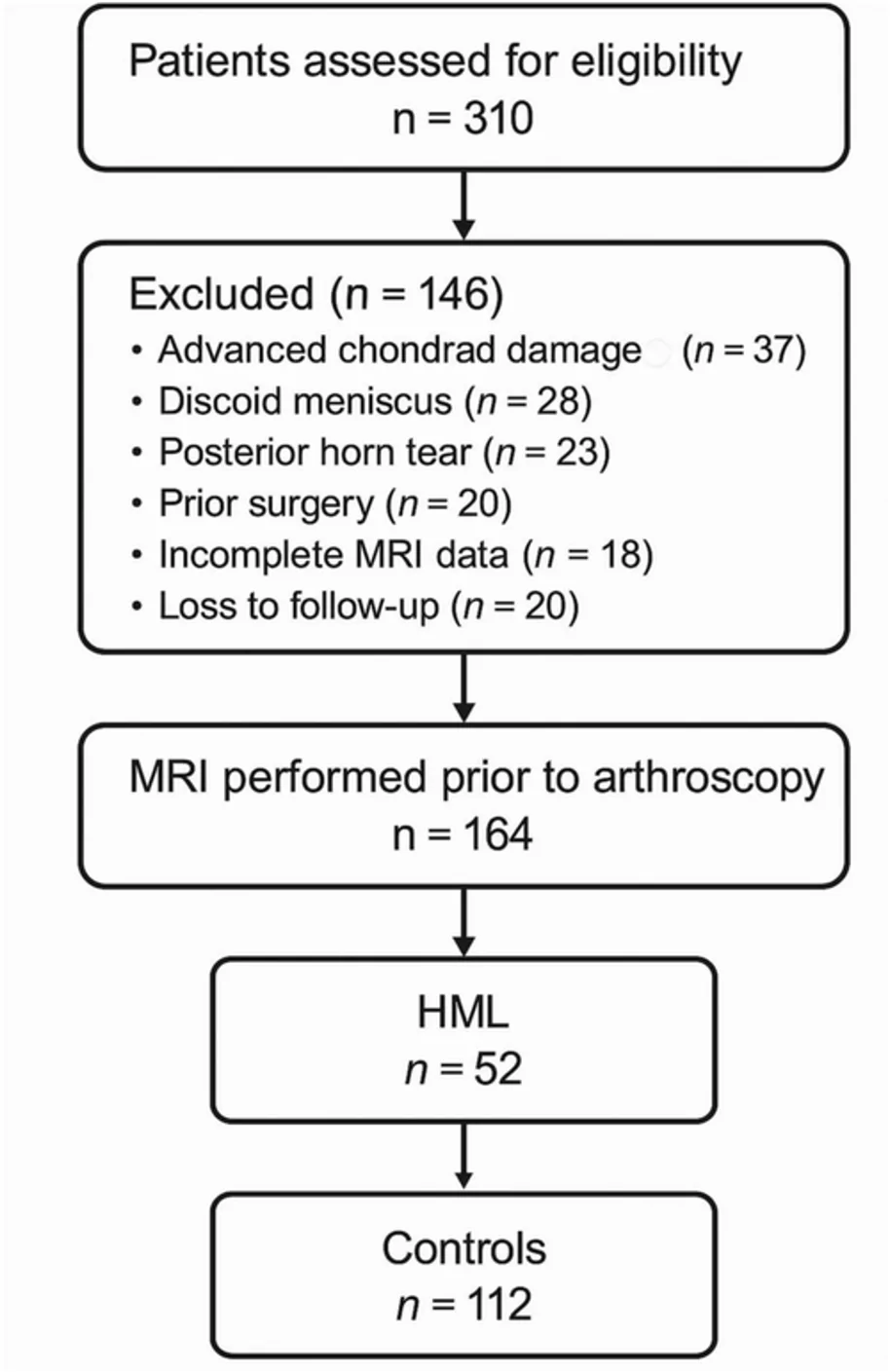
Study flow diagram. Flow diagram illustrating patient screening, eligibility assessment, exclusions and final inclusion for analysis. Of 214 MRI–arthroscopy pairs initially reviewed, 50 were excluded due to discoid morphology, prior lateral meniscal surgery, inadequate image quality or incomplete operative data, resulting in a final sample of 164 patients (52 with HML and 112 controls). HML, hypermobile lateral meniscus; MRI, magnetic resonance imaging.

Exclusion criteria were applied to avoid morphological variants or pathological conditions that could disrupt the native spatial relationship between the tibial spine and the posterior horn of the lateral meniscus. Patients were excluded if they exhibited a discoid or incomplete discoid lateral meniscus, radial or root tears or complex posterior horn configurations involving the posterior third of the meniscus. Prior lateral meniscal surgery or anterior cruciate ligament (ACL) reconstruction led to exclusion due to the potential for altered meniscotibial anatomy. Additional exclusion criteria included significant lateral compartment chondral injury (outerbridge Grade ≥3), acute tibial plateau fracture, congenital anomalies of the tibial spine or inadequate MRI quality due to motion or incomplete sequences. Patients with concomitant medial meniscal pathology were permitted as long as the lateral compartment fulfilled the inclusion criteria. All imaging studies were reviewed for sequence consistency and to confirm the absence of artefacts. Any patient lacking a clear operative description of posterior horn stability was excluded (Figure 1).

### MRI acquisition and image processing

All MRI examinations were performed using either a 1.5‐ or 3‐T scanner equipped with a dedicated knee coil. The institutional protocol included sagittal and coronal proton density–weighted sequences with and without fat suppression, supplemented by axial T2‐weighted images. Slice thickness ranged from 3 to 4 mm with an interslice gap not exceeding 0.5 mm. No specialized positioning, dynamic manoeuvres or loading devices were used; the objective was to determine whether TSMC could be measured reliably using routine MRI studies obtained in standard clinical settings. Images were exported in DICOM format and analysed using a calibrated workstation with digital measurement capabilities. All linear measurements were recorded in millimetres and reported to one decimal.

### TSMC measurement technique

**Figure 2:**
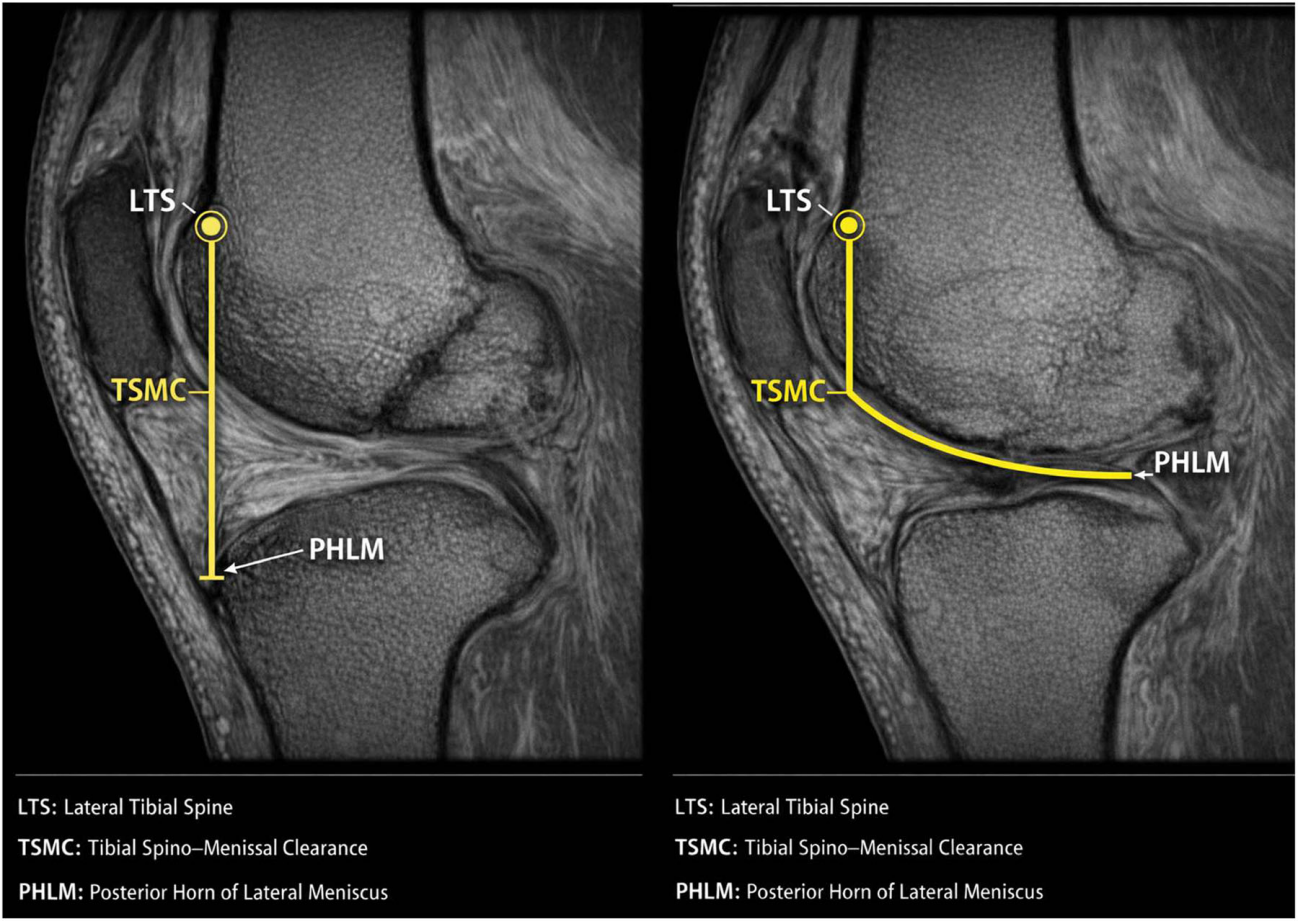
Measurement of TSMC. Sagittal proton density–weighted MRI demonstrating the measurement of TSMC. The sagittal slice showing maximal prominence of the lateral tibial spine is selected. A perpendicular line is drawn from the apex of the lateral tibial spine to the inner margin of the posterior horn of the lateral meniscus. The shortest linear distance between these landmarks is recorded as TSMC. LTS, lateral tibial spine; MRI, magnetic resonance imaging; PHLM, posterior horn of lateral meniscus; TSMC, tibial spino–meniscal clearance.

TSMC was defined as the shortest measurable linear distance between the apex of the lateral tibial spine and the inner border of the posterior horn of the lateral meniscus. Measurements were obtained from the sagittal slice that demonstrated maximal prominence of the lateral tibial spine. In nearly all cases, this slice corresponded to the midportion of the intercondylar eminence directly aligned with the posterior horn of the lateral meniscus. After selecting the appropriate slice, the observer identified the tibial plateau plane and drew a line perpendicular to its surface to minimize the influence of posterior tibial slope or variation in knee positioning. The perpendicular line was then extended posteriorly on the sagittal plane until it intersected the inner edge of the posterior horn. The shortest distance between the tibial spine apex and this intersection point was recorded as the TSMC value. If visualization of the posterior horn was partially obscured by artefact or volume averaging, the adjacent slice was examined; however, measurements were accepted only when the tibial spine remained sharply defined. Windowing adjustments were allowed, but any manipulation that could geometrically distort spatial relationships was strictly avoided. A schematic representation of the measurement procedure is shown in Figure 2 and Table 1.

**Table 1:** Practical guide for measuring TSMC.

1. Select the sagittal MRI slice showing maximal prominence of the lateral tibial spine.
2. Identify the apex of the LTS as the osseous reference point.
3. Draw a line perpendicular to the tibial plateau surface at the level of the spine apex.
4. Extend this line posteriorly on the sagittal plane until it intersects the inner margin of the PHLM.
5. Record the shortest linear distance between the spine apex and the PHLM in millimetres.

Guide for measuring TSMC			
Variable	HML (*n* = 52)	Controls (*n* = 112)	*p* Value
Age, years (mean ± SD)	29.4 ± 7.9	28.7 ± 8.3	0.62
Male sex, *n* (%)	31 (59.6)	63 (56.3)	0.70
Body mass index, kg/m^2^ (mean ± SD)	24.8 ± 3.1	24.5 ± 3.4	0.58
Right laterality, *n* (%)	26 (50.0)	59 (52.7)	0.75
Posterior tibial slope, ° (mean ± SD)	7.4 ± 1.9	7.6 ± 2.1	0.53
Lateral tibial plateau width, mm (mean ± SD)	34.1 ± 2.7	34.4 ± 2.8	0.49
MRI field strength 3.0 T, *n* (%)	21 (40.4)	48 (42.8)	0.77
Concomitant medial meniscal tear, *n* (%)	9 (17.3)	17 (15.1)	0.71

*Note*: TSMC can be measured on routine sagittal proton density–weighted MRI sequences without special positioning or dynamic imaging.

Abbreviations: HML, hypermobile lateral meniscus; LTS, lateral tibial spine; MRI, magnetic resonance imaging; PHLM, posterior horn of lateral meniscus; SD, standard deviation; TSMC, tibial spino–meniscal clearance.

### Observer protocol and reproducibility assessment

Three observers participated in the reproducibility analysis. Two were board‐certified musculoskeletal radiologists with at least seven years of experience interpreting knee MRI, and the third was an orthopaedic knee surgeon with expertise in meniscal pathology and imaging interpretation. All observers were blinded to patient identity, clinical presentation, arthroscopic findings and each other's measurements. Each observer measured TSMC twice, with at least a 2‐week washout interval to minimize recall bias. MRI studies were presented in randomized order for each measurement session.

Reproducibility was quantified using intraclass correlation coefficients (ICCs), calculated using a two‐way random‐effects model with absolute agreement. Intraobserver ICC values were derived from comparisons of repeated measurements by the same observer, and interobserver ICC values were based on the average of each observer's first measurements. ICC values were interpreted using conventional thresholds, with values above 0.90 considered indicative of excellent agreement. Bland–Altman plots were constructed to assess systematic bias and determine the limits of agreement for both intraobserver and interobserver pairs.

The orthopaedic knee surgeon involved as an observer did not participate in the surgical treatment of the patients included in the study and had no access to operative reports or arthroscopic findings.

### Arthroscopic assessment of meniscal stability

Arthroscopy was performed using standard anterolateral and anteromedial portals. Posterior horn stability was assessed using a probe pull‐test, in which posterior translation of the horn beyond the margin of the lateral tibial plateau was considered diagnostic of hypermobility. Dynamic probing during figure‐of‐four positioning was also used to assess excessive anteroposterior excursion. When abnormal displacement reproduced the same mechanical pattern described preoperatively—such as excessive posterior translation or engagement during dynamic probing—the meniscus was classified as hypermobile. All procedures were performed under spinal or general anaesthesia. Symptom reproduction was therefore interpreted in mechanical terms, based on abnormal meniscal excursion and engagement during probing manoeuvres, rather than patient‐reported pain. These criteria align with widely accepted definitions of HML in contemporary arthroscopic practice. In the retrospective cohort, operative reports were independently reviewed by two fellowship‐trained surgeons, with discrepancies resolved through consensus discussion. All arthroscopic evaluations were performed without access to MRI measurements.

### Clinical and anthropometric variables

The primary outcome was the presence or absence of arthroscopically confirmed HML. Secondary clinical variables collected included lateral joint‐line tenderness, history of mechanical symptoms, subjective episodes resembling pivot‐shift–type instability in ACL–intact knees and results of the meniscotibial stress test. In the prospective cohort, patient‐reported pain localized to the lateral compartment was recorded. Anthropometric covariates—age, sex, body mass index, tibial plateau width and posterior tibial slope—were extracted from clinical records and MRI measurements for potential inclusion in multivariable analyses.

### Statistical analysis

All statistical analyses were performed using SPSS (IBM Corp.) and R software (version 4.3). Distributional assumptions for continuous variables were evaluated using the Shapiro–Wilk test. Normally distributed variables were summarized using means and standard deviations, whereas non‐normally distributed variables were presented as medians with interquartile ranges. Categorical variables were summarized as frequencies and percentages. Between‐group comparisons of TSMC values were performed using independent‐samples *t* tests when parametric assumptions were met, or Mann–Whitney *U* tests when they were not. Effect sizes were calculated to quantify the magnitude of differences between groups.

Diagnostic performance was assessed using receiver operating characteristic (ROC) curve analysis. The area under the curve (AUC) was calculated along with 95% confidence intervals (CIs). The optimal cutoff point for TSMC was determined using the Youden index to maximize combined sensitivity and specificity. Additional diagnostic metrics included likelihood ratios and the diagnostic odds ratio (OR). To determine whether TSMC independently predicted HML, a multivariable logistic regression model was constructed that included age, sex, tibial plateau width, posterior tibial slope and MRI field strength. Variables with a univariable association of *p* < 0.10 were considered for inclusion. Model calibration was evaluated using the Hosmer–Lemeshow test and with calibration plots generated through 1000‐sample bootstrap resampling.

Reliability results were reported as ICC values with corresponding CIs. Bland–Altman plots were used to assess systematic bias and to determine the range of measurement differences considered acceptable. Statistical significance was defined as a two‐sided *p* value less than 0.05.

Sample size was determined based on diagnostic accuracy requirements rather than a priori hypothesis testing. Using previously reported recommendations for ROC analysis, a minimum of 10 outcome events per variable was required for multivariable logistic regression. Given the expected prevalence of HML and the planned number of covariates, a minimum sample of 150 MRI–arthroscopy pairs was considered adequate. The final cohort of 164 patients, therefore, provided sufficient power for reliable estimation of diagnostic performance and model stability.

### Risk of bias assessment

Methodological quality and risk of bias were assessed using the Quality Assessment of Diagnostic Accuracy Studies‐2 (QUADAS‐2) tool. The domains evaluated included patient selection, index test (TSMC measurement), reference standard (arthroscopic assessment) and flow and timing. Two independent reviewers performed the assessment, with discrepancies resolved by consensus.

## RESULTS

### Patient demographics and baseline characteristics

A total of 164 patients fulfilled the inclusion criteria and were incorporated into the final analysis. Among them, 52 patients (31.7%) exhibited arthroscopically confirmed HML, whereas 112 had stable posterior horns and served as controls. The mean age of the cohort was 28.9 ± 8.2 years, with no significant difference between the hypermobile group (29.4 ± 7.9 years) and controls (28.7 ± 8.3 years). The distribution of sex, body mass index and side of involvement did not vary meaningfully across groups. Anatomical variables—including posterior tibial slope and tibial plateau width—were also similar, and MRI characteristics such as field strength (1.5 vs. 3 T) were balanced. No patient had lateral compartment cartilage damage exceeding Outerbridge grade 2, and the prevalence of concomitant medial meniscal pathology did not differ significantly between groups. Overall, demographic and anatomical comparability between groups was maintained, minimizing potential confounding effects (Tables 1, 2 and 2).

**Table 2:** TSMC values and between‐group comparison.

Variable	HML (*n* = 52)	Controls (*n* = 112)	*p* Value
TSMC, mm (mean ± SD)	0.92 ± 0.28	1.68 ± 0.34	<0.001
TSMC < 1.2 mm, *n* (%)	46 (88.5%)	19 (17.0%)	<0.001
Effect size (Cohen's *d*)	—	2.51	—

Abbreviations: HML, hypermobile lateral meniscus; SD, standard deviation; TSMC, tibial spino–meniscal clearance.

**Table 3:** Interobserver and intraobserver reliability for TSMC.

Assessment	ICC	95% CI	Interpretation
Intraobserver—Observer 1	0.95	0.92–0.97	Excellent
Intraobserver—Observer 2	0.92	0.88–0.95	Excellent
Intraobserver—Observer 3	0.91	0.86–0.94	Excellent
Overall intraobserver ICC	0.93	0.89–0.96	Excellent
Interobserver ICC (three observers)	0.91	0.87–0.94	Excellent

Abbreviations: CI, confidence interval; ICC, intraclass correlation coefficient; TSMC, tibial spino–meniscal clearance.

### TSMC values in hypermobile and stable menisci

TSMC demonstrated a marked distinction between hypermobile and stable lateral menisci. Patients with HML had a mean TSMC of 0.92 ± 0.28 mm, significantly lower than the 1.68 ± 0.34 mm observed in controls (*p* < 0.001). The distribution curves showed minimal overlap: nearly all hypermobile cases measured below 1.2 mm, whereas most controls exceeded 1.4 mm. These differences were preserved when stratified by MRI magnet strength or slice thickness. Visual inspection of dispersion plots revealed consistent separation between groups regardless of posterior tibial slope or tibial plateau width, suggesting that TSMC represents a direct geometric marker of posterior horn position rather than a derivative of overall tibial morphology (Table 3).

**Table 4:** Multivariable logistic regression predicting hypermobile lateral meniscus.

Variable	Adjusted OR	95% CI	*p* Value
TSMC (per 0.1‐mm decrease)	5.6	3.2–9.9	<0.001
Age	1.01	0.97–1.05	0.62
Male sex	1.14	0.59–2.19	0.70
Posterior tibial slope	0.96	0.78–1.17	0.53
Lateral tibial plateau width	0.98	0.87–1.11	0.49
MRI 3.0 T (vs. 1.5 T)	1.08	0.56–2.10	0.77
Model AUC (bootstrap‐corrected)	0.89	—	—

Abbreviations: AUC, area under the curve; CI, confidence interval; OR, odds ratio; TSMC, tibial spino–meniscal clearance.

### Reproducibility of TSMC measurement

Reproducibility analysis confirmed excellent reliability. Intraobserver ICC ranged from 0.91 to 0.95, with a pooled intraobserver ICC of 0.93 (95% CI, 0.89–0.96). Interobserver agreement was similarly robust, with an ICC of 0.91 (95% CI, 0.87–0.94). Bland–Altman plots demonstrated negligible systematic bias, with mean differences approaching zero for all observer comparisons. The limits of agreement were narrow, particularly among the two radiologists, and no proportional bias was detected across the spectrum of TSMC values. These findings confirm that TSMC can be measured reliably in routine MRI studies by clinicians with different levels of imaging specialization (Tables 3 and 4).

### Diagnostic performance of TSMC

**Figure 3:**
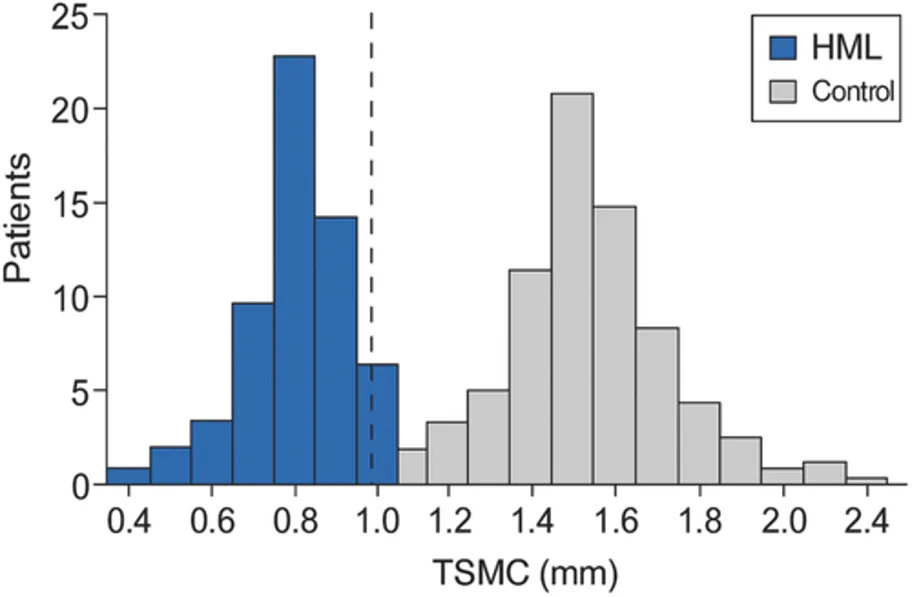
Distribution of TSMC Values in HML and control groups. Box‐and‐whisker plot comparing TSMC values between patients with HML and controls. HML cases cluster below the diagnostic threshold of 1.2 mm, whereas controls show substantially higher values. Minimal overlap is seen between groups, reflecting a clear separation of distributions. HML, hypermobile lateral meniscus; TSMC, tibial spino–meniscal clearance.

TSMC demonstrated strong discriminatory performance for identifying HML. The ROC curve yielded an AUC of 0.90 (95% CI, 0.85–0.94). The Youden index indicated an optimal diagnostic threshold of <1.2 mm. Using this cutoff, sensitivity reached 88.5% (95% CI, 78.3–95.0), while specificity was 83.0% (95% CI, 75.1–89.2). These values corresponded to a positive likelihood ratio of 5.2 and a negative likelihood ratio of 0.14, generating a diagnostic OR of 37.1. Subgroup analyses stratified by MRI field strength and patient sex showed consistent results, with AUC values ranging between 0.87 and 0.92. Calibration analysis revealed close concordance between predicted and observed probabilities, and the Hosmer–Lemeshow test confirmed adequate model fit (*p* = 0.71) (Figure 3).

### Influence of anatomical variables on TSMC

Further analyses assessed whether anatomical characteristics influenced TSMC values. Posterior tibial slope did not correlate with TSMC in either group, and tibial plateau width showed no association with clearance measurements. These findings underscore that TSMC reflects a specific spatial relationship pertinent to posterior horn stability rather than broader knee morphology.

### Multivariable logistic regression

**Figure 4:**
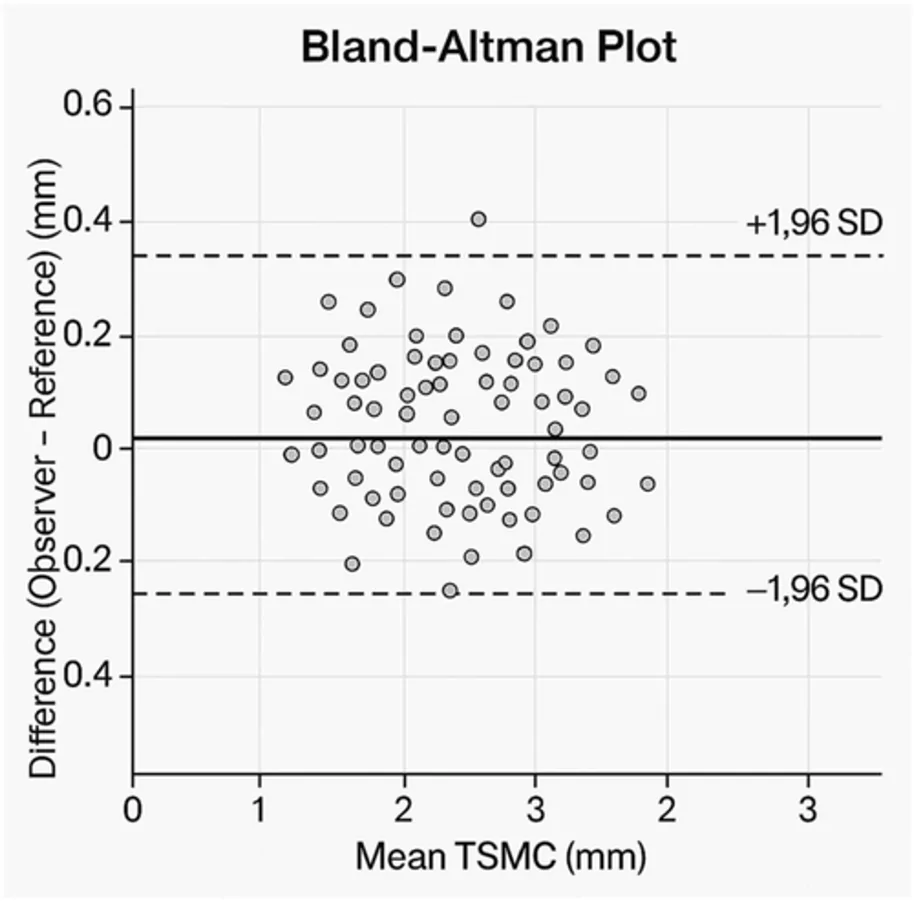
Bland–Altman Plots for TSMC Reliability. Bland–Altman plots assessing intraobserver and interobserver agreement for TSMC measurements. The mean bias is near zero, and the limits of agreement are narrow, indicating excellent reproducibility. No proportional bias is observed across the range of TSMC values. SD, standard deviation; TSMC, tibial spino–meniscal clearance.

**Figure 5:**
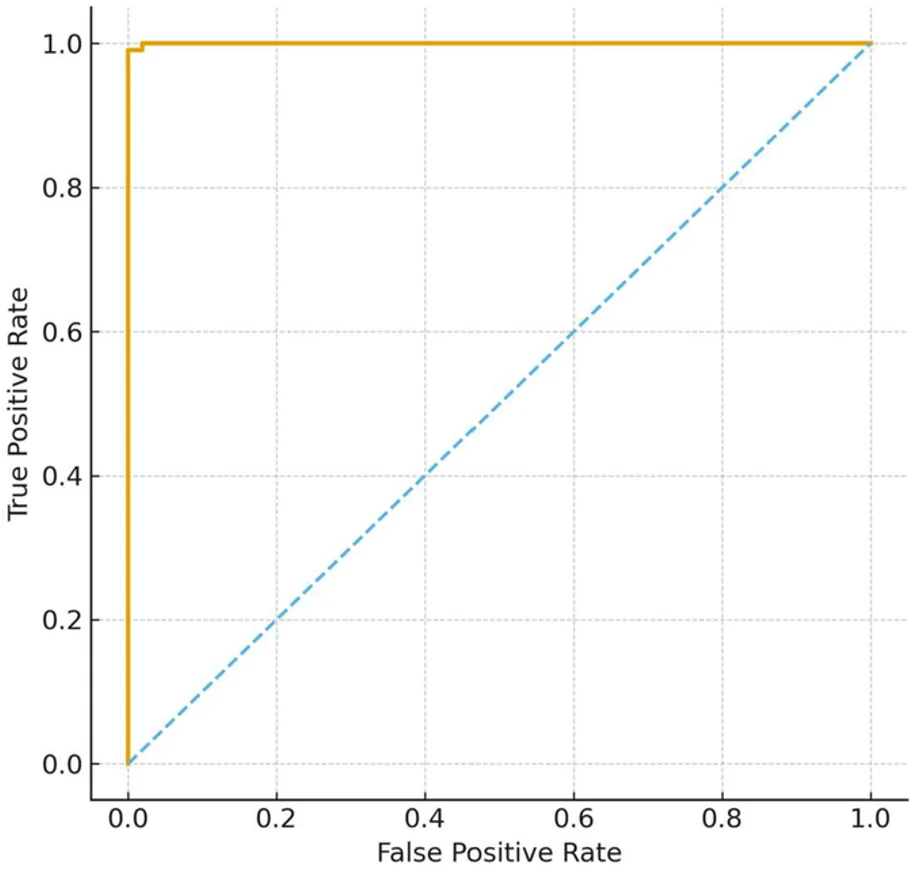
Receiver operating characteristic (ROC) curve for tibial spino–meniscal clearance (TSMC). ROC curve evaluating the diagnostic accuracy of TSMC for identifying hypermobile lateral meniscus. The area under the curve (AUC) is 0.90 (95% CI, 0.85–0.94). The optimal cutoff of <1.2 mm maximizes sensitivity and specificity for detecting instability. CI, confidence interval.

Multivariable regression analysis demonstrated that TSMC was an independent predictor of HML. After adjusting for age, sex, tibial plateau width, posterior tibial slope and MRI field strength, decreasing TSMC remained strongly associated with hypermobility. Each 0.1‐mm reduction in TSMC increased the odds of HML (adjusted OR, 5.6; 95% CI, 3.2–9.9; *p* < 0.001). No other covariate reached statistical significance, indicating that the predictive value of TSMC was robust and not confounded by demographic or anatomical variability. Following bootstrap validation, the corrected AUC of the multivariable model was 0.89, confirming strong overall discriminative ability (Figures 4, 5 and 5).

### Clinical correlates and secondary outcomes

Secondary clinical findings corresponded with TSMC values. Patients with arthroscopically confirmed hypermobility reported a greater frequency of lateral mechanical symptoms and more consistent joint‐line tenderness. Among the prospective participants, those classified as hypermobile described higher lateral pain scores at initial evaluation. Despite these associations, none of the clinical variables independently predicted hypermobility once TSMC was incorporated into the regression model, suggesting that quantitative MRI assessment provides diagnostic precision beyond that achievable through clinical examination alone. Pivot‐shift–like sensations in the ACL–intact knees were more common among hypermobile subjects but did not influence the multivariable model.

## DISCUSSION

### Main findings

The present study evaluated TSMC as an MRI‐derived imaging measurement associated with arthroscopically confirmed HML. Lower TSMC values were observed in hypermobile compared with stable lateral menisci, and the measurement demonstrated high inter‐ and intraobserver agreement. These findings suggest that the spatial relationship between the posterior horn of the lateral meniscus and the lateral tibial spine may be associated with functional meniscal instability. However, these results should be interpreted as exploratory and require further validation before routine clinical implementation [2, 3, 11, 12, 13, 14, 15].

### Biomechanical interpretation

From a biomechanical perspective, the posterior horn of the lateral meniscus is stabilized by meniscotibial and capsular attachments that limit excessive anteroposterior excursion. Laxity of these structures, even in the absence of a discrete tear, may allow abnormal posterior horn displacement that can be reflected as a reduced clearance relative to the tibial spine on sagittal MRI. This interpretation is consistent with prior anatomical and biomechanical studies describing the stabilizing role of the posterolateral meniscotibial complex and the contribution of posterior horn fixation to rotational knee stability. Accordingly, TSMC may capture a geometric manifestation of functional instability rather than secondary morphological variation [4, 6, 16, 17, 18, 19, 20, 21].

### Comparison with existing literature

Previous approaches to imaging assessment of lateral meniscal instability have largely relied on indirect or qualitative MRI findings, such as subtle posterior horn displacement or perimeniscular fluid, which have demonstrated inconsistent diagnostic performance. Consequently, the diagnosis of HML has traditionally depended on dynamic arthroscopic probing, as conventional MRI often fails to identify instability in structurally intact menisci. In this context, TSMC differs from previously proposed imaging features by providing a simple linear measurement derived from routine MRI sequences. Nevertheless, it should be regarded as a complementary imaging measurement rather than a replacement for arthroscopic evaluation, particularly given the absence of external validation at this stage [5, 16, 17, 18, 19, 20, 21, 24].

### Clinical implications

Accurate preoperative suspicion of HML may assist in surgical planning, particularly when meniscal preservation is considered. In the absence of reliable imaging criteria, lateral meniscal hypermobility is frequently identified only at arthroscopy, which may lead to unplanned procedures or partial meniscectomy in cases where stabilization could be more appropriate. If confirmed in future studies, TSMC may help identify patients in whom targeted meniscal stabilization techniques should be anticipated. At present, however, this imaging measurement should be interpreted cautiously and in conjunction with clinical findings and intraoperative assessment [6, 9, 10, 20, 21, 22, 23, 25, 26].

Unnecessary lateral meniscectomy, even when limited, has been associated with increased compartmental contact pressures and an elevated risk of early degenerative changes. Misinterpretation of hypermobility as a structural tear may therefore result in avoidable resection of otherwise salvageable tissue. By potentially contributing to improved preoperative suspicion of instability, TSMC may support a shift toward meniscal‐preserving strategies. Nonetheless, the proposed cutoff value should not be considered definitive and requires confirmation in independent cohorts before incorporation into diagnostic algorithms [7, 25, 26, 27, 28].

### Strengths of the study

Several methodological aspects strengthen the present investigation. The TSMC measurement demonstrated high reproducibility across observers with different clinical backgrounds, suggesting that it can be applied without reliance on specialized radiologic expertise. Arthroscopy was used as the reference standard, ensuring that imaging findings were compared against direct assessment of meniscal stability. In addition, consistent performance across MRI field strengths and anatomical subgroups supports the internal robustness of the measurement. These strengths support the feasibility of TSMC as an MRI‐derived imaging measurement while underscoring the need for further confirmation [3, 5, 28].

### Limitations

This study has several limitations. Its single‐centre design may limit external generalizability, and validation in multicenter settings with varied imaging protocols is required. Although a standardized MRI protocol was used, variability related to slice orientation, sequence parameters and patient positioning may influence measurement accuracy in routine practice. The exclusion of patients with advanced chondral disease or complex meniscal morphology, while methodologically justified, may restrict applicability to broader clinical populations. Finally, clinical outcomes were not assessed; therefore, the prognostic value of TSMC in predicting postoperative symptom resolution or recurrence of instability remains unknown. These aspects should be addressed in future investigations [28].

## CONCLUSION

TSMC is a simple, reproducible MRI measurement that reliably identifies HML. Lower TSMC values were strongly associated with arthroscopically confirmed instability and demonstrated excellent inter‐ and intraobserver reliability. Because TSMC can be obtained from routine MRI without additional sequences or positioning, it represents a practical preoperative biomarker to support the diagnosis of lateral meniscal hypermobility and guide meniscal‐preserving surgical decision‐making.

## AUTHOR CONTRIBUTIONS

Camilo Helito contributed to the conception of the study, development of the MRI measurement protocol and critical revision of the manuscript for important intellectual content. He supervised methodological accuracy and ensured consistency in imaging interpretation. Francisco Endara Urresta participated in study design, coordinated patient recruitment and arthroscopic data collection, performed data analysis and interpretation and drafted the initial version of the manuscript. He also contributed to revisions and final approval of the submitted work. Carlos Peñaherrera‐Carrillo contributed to MRI acquisition review, reproducibility assessment, statistical validation and refinement of the diagnostic methodology. He critically revised the manuscript and approved the final version. Alejandro Barros Castro assisted with data extraction, MRI measurements, observer reliability analysis and preparation of figures and tables. He contributed to manuscript editing and approved the final submitted version. All authors read and approved the final manuscript and agree to be accountable for all aspects of the work.

## CONFLICT OF INTEREST STATEMENT

The authors declare no conflict of interest.

## ETHICS STATEMENT

Institutional ethics approval was obtained from Clinica Arthros (ARTC‐0044).

## Data Availability

The authors have nothing to report.
